# Inhibition of Cell Proliferation in an NRAS Mutant Melanoma Cell Line by Combining Sorafenib and α-Mangostin

**DOI:** 10.1371/journal.pone.0155217

**Published:** 2016-05-06

**Authors:** Yun Xia, Ying Li, Kenneth D. Westover, Jiaming Sun, Hongxiang Chen, Jianming Zhang, David E. Fisher

**Affiliations:** 1 Department of Plastic Surgery, Union Hospital, Tongji Medical College, Huazhong University of Science and Technology, Wuhan, Hubei, China; 2 Cutaneous Biology Research Center, Massachusetts General Hospital, 149 Building 13th ST, Charlestown, Massachusetts, United States of America; 3 Clinical Laboratory, The Third Affiliated Hospital of Zhengzhou University, No.7 Front Kangfu ST, Zhengzhou 450052, China; 4 Departments of Biochemistry and Radiation Oncology, University of Texas Southwestern Medical Center at Dallas, Dallas, Texas, United States of America; Rutgers University, UNITED STATES

## Abstract

α-Mangostin is a natural product commonly used in Asia for cosmetic and medicinal applications including topical treatment of acne and skin cancer. Towards finding new pharmacological strategies that overcome NRAS mutant melanoma, we performed a cell proliferation-based combination screen using a collection of well-characterized small molecule kinase inhibitors and α-Mangostin. We found that α-Mangostin significantly enhances Sorafenib pharmacological efficacy against an NRAS mutant melanoma cell line. The synergistic effects of α-Mangostin and Sorafenib were associated with enhanced inhibition of activated AKT and ERK, induced ER stress, and reduced autophagy, eventually leading to apoptosis. The structure of α-Mangostin resembles several inhibitors of the Retinoid X receptor (RXR). MITF expression, which is regulated by RXR, was modulated by α-Mangostin. Molecular docking revealed that α-Mangostin can be accommodated by the ligand binding pocket of RXR and may thereby compete with RXR-mediated control of MITF expression. In summary, these data demonstrate an unanticipated synergy between α-Mangostin and sorafenib, with mechanistic actions that convert a known safe natural product to a candidate combinatorial therapeutic agent.

## Introduction

Melanoma is a common, deadly form of skin cancer associated with sun exposure whose incidence has been on the rise in recent years [1]. Historically melanoma has been insensitive to systemic therapies and the primary treatment was surgical excision. BRAF and NRAS mutations are the most common genetic drivers of melanoma, found in ~50% and ~20% of tumors respectively, and are nearly mutually exclusive [2,3]. Several targeted therapies are now available for BRAF-mutated melanoma [4,5]. However there are few effective targeted therapy options for RAS-driven cancers. Identification of targetable vulnerabilities in NRAS mutated cancers is an area of active research but so far no effective single-agent systemic therapies have been identified [6,7]. Based on the observation that signaling pathways downstream of NRAS often interact through ‘cross talk’ or feedback loops, attention has turned to identifying combinations of drugs targeting effectors involved in distinct RAS signaling pathway [8].

Here we have examined a potential use of α-Mangostin, a naturally occurring organic xanthonoid compound which can be isolated from various parts of the mangosteen tree (*Garcinia mangostana*), as a potential combinatorial agent against an NRAS mutated melanoma cell line. Xanthones extracted from mangosteen have shown anti-proliferative activity in human cancer cell lines, such as leukemia, breast cancer and liver cancer [9–11]. Isolates of α-Mangostin are widely used in Southeast Asia for medicinal purposes based on antioxidant, anti-bacterial, anti-inflammatory, anti-cancer and skin cancer prevention activities. Previously we found α-Mangostin to have limited anti-proliferation activities against human primary melanocytes at up to 10 μM concentration [12]. We reasoned that α-Mangostin might exhibit combinatorial activity when paired with kinase inhibitory small molecules, which might reveal synergistic opportunities. To this end, we performed a combination screen using a collection of well-characterized kinase inhibitors whose targets are known, against the NRAS-mutant melanoma cell line, SK-MEL2.

## Materials and Methods

### Combination screening of kinase inhibitor library with α-Mangostin

The library of kinase inhibitors (Table A in S1 File) was screened against the SK-MEL-2 cell line alone or in combination with α-Mangostin to test for compounds with synergistic effects. Cells were plated in 384-well clear bottom plates (Corning, Tewksbury, MA, USA) with 20,000 cell/ml density. Kinase inhibitors were pin-transferred into each well at a concentration of 2 μM with or without 5 μM α-Mangostin. Cell proliferation and cytotoxicity were measured via CellTiter-Glo Luminescent Cell Viability Assay Kit (Promega, Madison, WI) at 48 hours. The combination index (CI) was used to quantitatively define an additive effect (CI = 1), synergism (CI < 1), and antagonism (CI > 1) in drug combinations. CI was calculated as described in (Chou 1984), using Calcusyn software (Biosoft, UK) [13].

### Human melanoma cell lines culture

SK-MEL-2 cells and SK-MEL-30 cells were grown in Dulbecco’s modified Eagle medium (DMEM; fetal calf serum, 10%; penicillin, 100 000 U/l; streptomycin sulphate, 100 mg/l; Life Technologies, Grand Island, NY, USA). UACC257 cells were grown in Roswell Park Memorial Institute (RPMI) 1640 Medium (serum, 10%; penicillin, 100 000 U/l; streptomycin sulphate, 100 mg/l; Life Technologies, Grand Island, NY, USA). Human primary melanocytes were grown in TIVA Medium (Hams F10; fetal bovine serum 10%; Pennicillan 10,000 U/ml; Streptomycin 10,000 U/ml; Glutamine 29.2 mg/ml; TPA-Phorbal Myristate Acetate 100 μg/ml; 3-Isobutyl-1-Methylxanthine 5x 10^-3^ M; N^6^, 2′-O-Dibutyryladenosine 3′,5′-cyclic monophosphate sodium salt 1 x 10^-2^ M; Sodium Orthovanadate 100 mM; Fungizone 250 μg/ml; Geneticin 50 mg/ml; Gibco and Sigma). Cells were incubated at 37°C in 5% CO_2_, 5% humidify and passaged at 2 x 10^4^ cells/ml when near-confluent monolayers were achieved. Cells were verified as free from Mycoplasma contamination.

### Treatment of melanoma cells

The pan-RAF inhibitor Sorafenib was purchased from Bayer Corporation, West Haven, CT, and α-Mangostin was purchased from Sigma. Compounds were dissolved in DMSO and added directly to the culture medium of melanoma cells at the concentrations to be tested. Melanoma cells incubated with culture medium with vehicle (DMSO) served as controls.

### Cell proliferation assay

Drug cytotoxicity was determined using CellTiter-Glo Luminescent Cell Viability Assay Kit (Promega, Madison, WI). Cells were plated in 96-well clear bottom plates (Corning, Tewksbury, MA, USA) at a density of 20 000 cells/ml in culture medium (DMEM supplemented with 10% FBS and penicillin/streptomycin), drugs were added to the first well of each row at a concentration of 2 μM alone or in combination with α-Mangostin (5 μM), then double diluted more than 10 times. After 48 hours of culture, CellTiter-Glo reagents were used to measure cell proliferation ratio. Luminescent assay measurements were read on the EnVision^®^ Multilabel Plate Reader (Envision, Perkin Elmer) using ultrasensitive luminescence.

### Colony formation assay

SK-MEL-2 cells were seeded in 6-well plates at a density of 100 cells/well and drugs were added into each well at a concentration of 5 μM alone or with α-Mangostin (2 μM) for 6 days. Cells were fixed with 4% paraformaldehyde, and colonies were stained with 0.1% crystal violet.

### Immunofluorescence microscopy

SK-MEL-2 cells mounted on glass slides were fixed with 4% paraformaldehyde for 20 minutes, and permeabilized with PBS containing 0.1% Triton X-100 and 0.1% glycine for 2 minutes on ice. TUNEL Staining was performed using In Situ Cell Death Detection Kit, Fluorescein (Roche Diagnostics, Germany). Cells were co-stained with 4’6’-diamidino-2-phenylindole (DAPI) to visualize nuclei. The fluorescence images were taken using a FSX100 all-in-one microscope (Olympus Corporation, Japan)

### Western blotting

Western Blot analysis was performed as described previously [14]. The following primary antibodies were used: anti-ERK, anti-phospho-ERK (Thr202/Tyr204), anti-AKT, anti-phospho- AKT (Thr473), anti-Mcl-1, anti-beta-actin, anti-phospho-eIF2a, anti-eIF2a, anti-LC3, anti-Atg5, anti-MITF (Cell Signaling Technology). UACC257 cells were cultured in Vitamin D deficient medium for more than one week, and then treated with different concentration of α-Mangostin (5 μM or 0.5 μM), ATRA (5 μM or 0.5 μM) or their combination for 8 hours. The expression level of MITF was detected by western blot. ChemiDoc XRS system (Bio-Rad) was used for imaging.

### RT-PCR of CHOP and 36B4 relative expression

SK-MEL-2 cells were treated with α-Mangostin (2 μM), Sorafenib (5 μM) or their combination for 8 hours. Total RNA was extracted from treated or vehicle control (DMSO treated) cells by using an RNeasy kit (QIAGEN). The RNAs were PCR amplified using specific primers for CHOP and 36B4. The primers used were CHOP forward (5’-GCGTCTAGA TCATGCTTGGTGCAGATTC-3’), CHOP reverse (5’-GCGTCTAGAATGG CAGCTGAGTCATTGCC-3’), to yield a 509-bp amplicon. The primers for the 36B4 RNA, which served as an internal standard, were obtained from Life Technologies.

### Statistical and computational analysis

The significance of the differences between the experimental conditions was determined using Student’s t test for unpaired observations.

Drug Interaction analysis: The software Calcusyn (Biosoft, UK) was used for calculating drug combination effect. CI (Combination Index) was used as the indicator of drug combining dose-effect.

Molecular docking: Docking was performed using the Glide program within the Schrödinger software suite. Coordinates for PDB ID 3A9E [15] were loaded and prepared using the Protein Preparation Wizard by addition of hydrogen atoms and elimination of non-interacting waters. A docking grid was generated for the region surrounding the RXR ligand, (2E, 4E, 6Z)-3- methyl-7- (5,5,8,8-tetramethyl-3-propoxy-5,6,7,8-tetrahydronaphthalen-2-yl)octa-2, 4,6-trienoic acid, in the original structure without imposing positional or hydrogen bond constraints. Ligands were prepared using LigPrep for all possible states between pH 5 to pH 9. During docking both rigid and flexible ligand sampling were allowed and the top scoring poses were evaluated.

## Results

### Screening of the kinase inhibitors and verification of the combination effect

**Fig 1:**
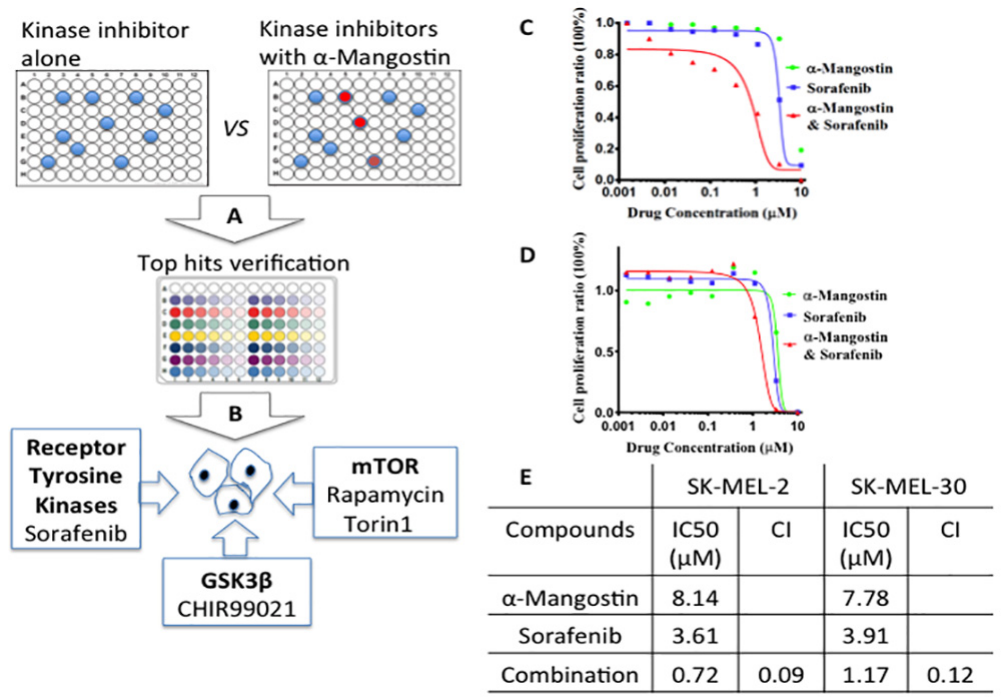
A cell proliferation based combination screening of α-Mangostin with kinase specific inhibitors. A and B, show the screening scheme of primary screening and hit identification on SK-MEL-2 cell line. C, The dosage dependent cell proliferation assay of drugs and drug combination on SK-MEL-2 cells. D, The dosage dependent cell proliferation assay of drugs and drug combination on SK-MEL-30 cells. E, Synergistic effect of drug combination. CI (Combination Index) value at half lethal dosage IC_50_ indicated a strong synergistic effect of α-Mangostin and Sorafenib combination.

To search for potential synergistic effects of combinatorial inhibition by α-Mangostin and kinase inhibitors, a collection of known kinase inhibitors was screened with or without the presence of α-Mangostin, which alone showed quite mild cytotoxity with IC50 >10 μM in human primary melanocytes (Table B in S1 File). The screening scheme is illustrated in Fig 1A and 1B and utilizes a cell proliferation assay in the NRAS mutant melanoma cell line SK-MEL-2. Top hit compounds were identified as significantly inhibiting cell proliferation after being combined with α-Mangostin at a concentration of 5 μM and further verified by dose-response relationships profiling assay. Analysis of validated hits indicated a relatively narrow clustering of targets which included the inhibitors of the receptor tyrosine kinases VEGFR, EGFR, mTOR, and GSK3β (Fig 1B). Sorafenib, Rapamycin, Torin1 and CHIR99021 are representative compounds.

We were specifically interested in the combinatorial effect of Sorafenib and α-Mangostin since Sorafenib has already been FDA approved for use in humans; current indications are for advanced renal cell and hepatocellular carcinomas. Although Sorafenib has shown little promise as a single agent in melanoma patients, Sorafenib targets multiple kinases including VEGFR, PDGFR, FLT3, C-Kit and the RAF family [16]. In a 48 hour cell proliferation assay, α-Mangostin enhanced Sorafenib potency more than 5 fold in SK-MEL-2 cells by improving Sorafenib’s IC_50_ from 3.41 μM to 0.71 μM (Fig 1C and 1E), and more than 3 fold in SK-MEL-30 cells from 3.9 μM to 1.2 μM (Fig 1D and 1E). The synergistic effect of α-Mangostin and Sorafenib was further verified with Calcusy analysis [13]. The Combination Index (CI) is widely used as an indicator of drug interactions. The quantitative definition of additive effect (CI = 1), synergism (CI < 1), and antagonism (CI > 1) were used in drug combinations. When calculated for α-Mangostin and Sorafenib, the CI was 0.1 both in SK-MEL-2 cells and SK-MEL-30 cells at the half lethal dosage, suggesting strong synergistic effects (Fig 1E).

### Combining α-Mangostin and Sorafenib suppresses cell colony formation

**Fig 2:**
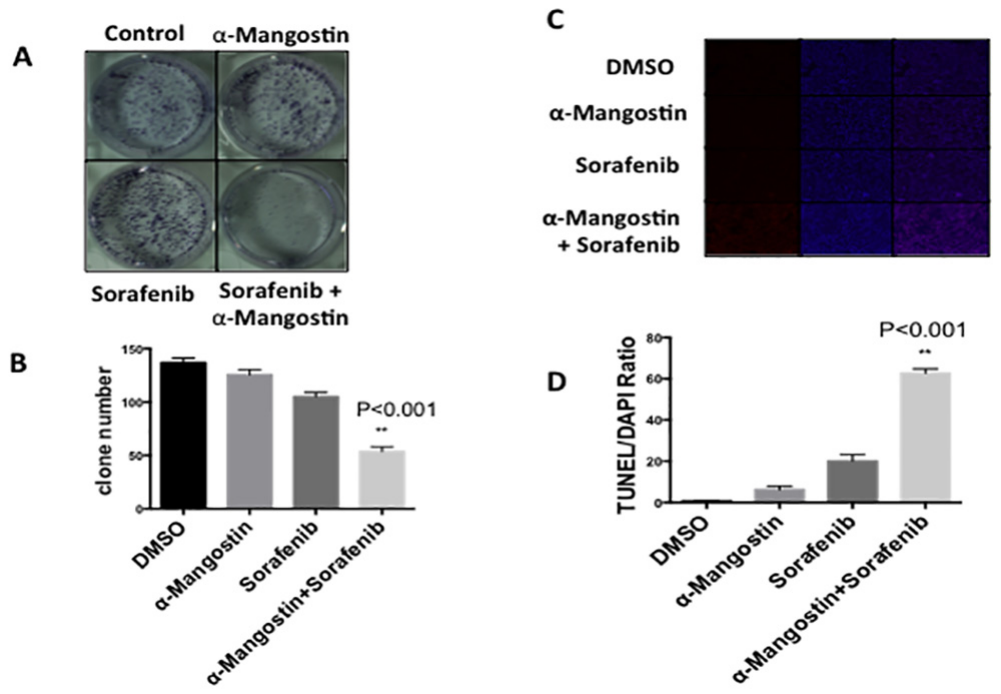
α-Mangostin activates Sorafenib induced apoptosis. A, Clonogenic survival assay was performed to examine the synergistic effect of the combination of Sorafenib and α-Mangostin on the survival of SK-MEL-2 cells. Cell colonies ware stained in blue. B, Colony numbers were counted. The combination of Sorafenib and α-Mangostin significantly suppressed colony formation (p = 0.001). C, TUNEL staining detects cell apoptosis. Slices were cultured in medium containing Sorafenib (5 μM), α-Mangostin (2 μM) or their combination for 3 hours and stained for TUNEL (red) or 4′, 6-diamidino-2-phenylindole (DAPI; blue). Representative images of three independent experiments are shown. D. Apoptotic cells were counted. Student’s T-test showed that a significant increase of apoptotic cells appeared in the drug combinatorial treatment (p = 0.0003).

**Fig 3:**
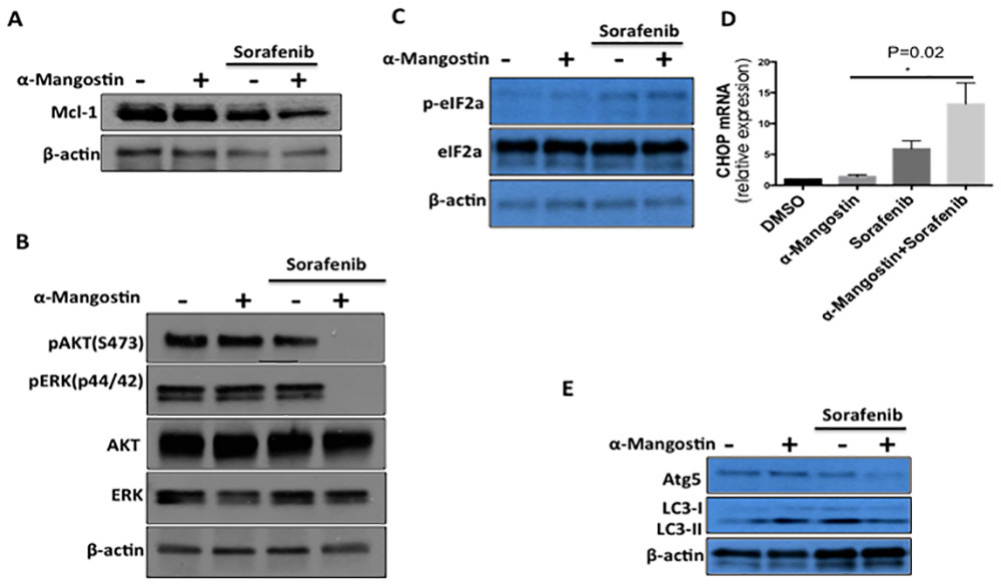
Impact of Sorafenib and α-Mangostin combination on AKT, ERK, and ER stress and autophagy markers. A, MCL-1 expression was suppressed by Sorafenib (5 μM) and α-Mangostin (2 μM) treatment for 8 hours in SK-MEL-2 cells. B, Down regulation of phosphorylation of AKT and ERK is much stronger at three hours treatment with the combination of Sorafenib (5 μM) and α-Mangostin (2 μM) as compared to single agent treatments. C, The increased expression of phosphorylation of eIF2α were induced by the combination of Sorafenib and α-Mangostin for 8 hours, indicating ER stress. D, The relative mRNA expression level of CHOP. SK-MEL-2 cells were treated with Sorafenib (2 μM), α-Mangostin (2 μM) or their combination for 3 hours. The relative mRNA expression level of CHOP was detected by real-time polymerase chain reaction (PCR). Data shown are the means (±SEM) from at least three independent experiments. The statistical significance increase of CHOP expression was determined by Student’s T test (P = 0.02). E. Atg5 and LC3II levels were detected after the treatment of Sorefenib (5 μM) or α-Mangostin (2 μM).

We next asked if the synergistic effect of Sorafenib and α-Mangostin on inhibition of SK-MEL-2 could be replicated in prolonged cell culture and therefore performed a 6-day clonogenic growth assay. SK-MEL-2 cells treated with Sorafenib (5 μM) and α-Mangostin (2 μM) significantly suppressed colony growth by more than 50%, as compared to cells grown in the presence of individual agents (5 μM Sorafenib or 2 μM α-Mangostin) (Fig 2A and 2B). We then asked whether the combination of Sorafenib and α-Mangostin was inducing apoptosis in these cancer cells using TUNEL (Terminal deoxynucleotidyl transferase dUTP nick end labeling) staining assays. Strong TUNEL staining indicates DNA fragmentation and activated apoptosis. We saw a significant increase in the number of apoptotic cells when Sorafenib was combined with α-Mangostin treatment (Fig 2C and 2D). To further examine this observation, we monitored levels of Mcl-1, an important component of many survival pathways [17], in prolonged 8 hour drug treatment experiments and noted a significant down regulation with combination treatment (Fig 3A). We also observed increased levels of cleaved-PARP for the combination treatment (Figure A in S1 File). These results suggest that apoptosis could play a major role in the enhanced anti-proliferative effects of Sorafenib/α-Mangostin treatment on SK-MEL-2 cells.

### The combination effect of α-Mangostin and Sorafenib on AKT and ERK activation

To explore signaling consequences upon exposure to combination Sorafenib and α-Mangostin, we examined the expression and activation status of AKT and ERK which are often activated in RAS-driven tumors and are cytoprotective, as has been observed in drug-resistant melanoma [18,19]. The effects of Sorafenib (5 μM), α-Mangostin (2 μM) or their combination on the phosphorylation of AKT and ERK in SK-MEL-2 cells for 3 hours were analyzed by Western blot (Fig 3B) and revealed that the phosphorylation of AKT (S473) and ERK are more strongly suppressed with combination treatment when compared to single agent treatment.

### α-Mangostin enhances Sorefenib’s induction of ER stress and inhibits autophagy

Sorafenib has been shown to induce autophagy in human hepatocellular carcinoma cells (HCC) through a mechanism that involves endoplasmic reticulum (ER) stress [20]. Autophagy upregulation has also been proposed as a possible mechanism for drug resistance in HCC [20]. To evaluate whether similar mechanisms may be involved here, we studied markers of autophagy and ER stress. Expression levels of CHOP and phosphorylation levels of eIF2-α are prototypical markers of the endoplasmic reticulum stress dependent unfolded protein response (UPR) signaling pathway [21]. Autophagy protein 5 (Atg5), an E3 ubiquitin ligase, is required for autophagy due to its role in autophagosome elongation and conversion of the soluble form of LC3 (LC3-I) to a lapidated and autophagosome-associated form (LC3-II) [22]. We found elevated expression levels of CHOP and p-eIF2α in SK-MEL-2 cells upon combinatorial treatment with α-Mangostin and Sorefenib (Fig 3C and 3D) suggesting that ER stress and autophagy could induce apoptosis. However, expression levels of autophagy-related proteins Atg5 and LC3II (Fig 3C and 3D) were decreased in SK-MEL-2 cells treated with the combination of 5 μM Sorefenib and 2 μM α-Mangostin for 8 hours. These data suggest that the relationship of this drug combination to autophagy is not straightforward and may or may not contribute to the mechanism of synergy.

### α-Mangostin Acts Through the Retinoic Acid Receptor (RXR)

**Fig 4:**
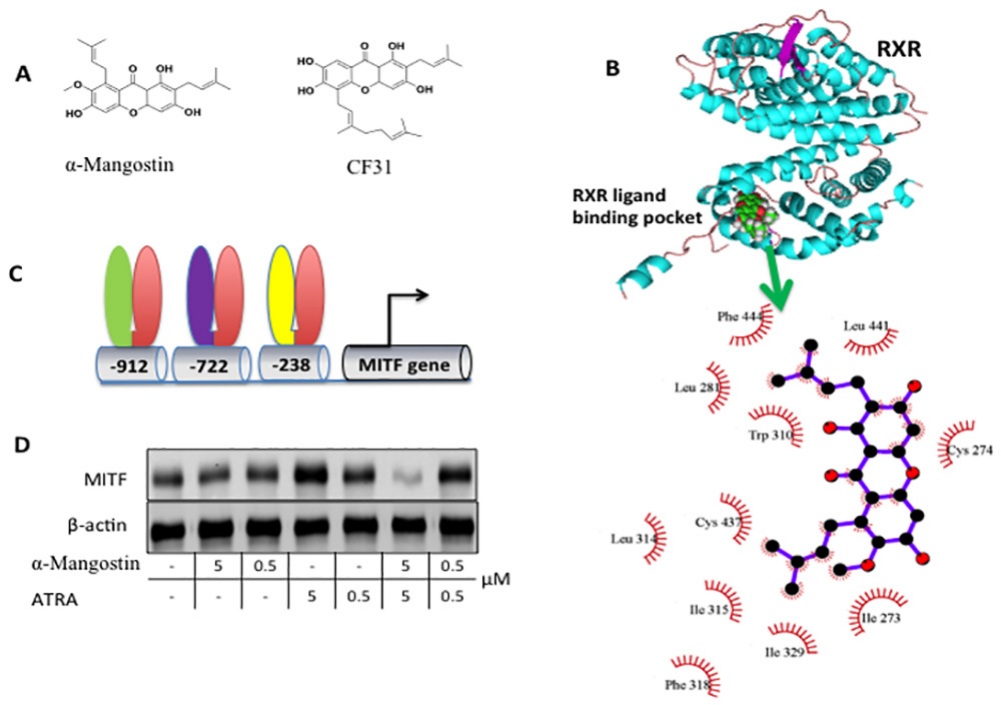
α-Mangostin modulates MITF expression via RXR binding. A. Chemical structure of α-Mangostin resembles CF31, which targets RXR. B, Molecular docking shows that α-Mangostin is accommodated by the RXR ligand binding pocket. In the top panel RXR is cyan and α-Mangostin is shown as spheres. A close-up view of interactions between RXR and α-Mangostin is shown in the bottom. C. Structure of MITF promoter region. The binding sites of VDR/RXR, RAR/RXR D5 and NURR1/RXR are located at position of -912, -722, and -238 respectively. D. ATRA, a RAR activator, significantly induces expression of MIFT in a dose dependent manner but α-Mangostin blocks expression.

To gain further clues regarding the molecular mechanism(s) of action for α-Mangostin, we used a compound-structure oriented approach to identify possible targets of α-Mangostin (Fig 4A). We searched for compounds with known biological targets whose core structures were similar to α-Mangostin and found several including RXR modulators LG100754 [23] and CF31 [24]. The binding of CF31 and RXR- α results in inhibition of RXR- α transactivation and induces TNF- α dependent cancer cell apoptosis. Additionally, we previously reported that α-Mangostin could alter MITF (Microphthalmia-associated transcription factor) mRNA expression [12,25], which has been suggested to be regulated by RAR (Retinoic Acid Receptor) and VDR (Vitamin D Receptor) by forming a heterodimer with RXR (Retinoid X Receptor)[26–28].

A molecular docking study using a previously reported crystal structure of RXR (PDB 3A9E) showed that α-Mangostin is compatible with the RXR’s ligand binding pocket, the common binding partner of RAR and VDR (Fig 4B). Comparison of a docking model of α-Mangostin and several analogues to the previously characterized binding mode of the RXR ligand LG100754 [15,24] showed similar modes of insertion between LG100754, α-Mangostin and its analogs into the RXR hydrophobic ligand binding cleft. However, stabilization of the interaction of α-Mangostin and RXR protein is primarily by hydrophobic interactions (Fig 4B).

Based on the docking results and similarity to other RXR ligands, we hypothesized that α-Mangostin might interrupt RAR and VDR binding through binding to the RXR ligand binding pocket. To test this, we studied the effects of α-Mangostin on UACC 257 cells, where it had been previously shown that the expression level of MITF is regulated by RXR and associated proteins [29]. More specifically, motif mapping of the MITF promoter suggested that a VDR/RXR binding motif is located at position -912, and a RAR/RXR D5 binding motif is at position -722. MITF also has a NURR1/RXR binding site at -238 (Fig 4C). Western analysis confirmed that α-Mangostin modulates MITF expression in a dose dependent manner, and the upregulation of MITF expression level by ATRA is neutralized by 5 μM α-Mangostin (Fig 4D).

## Discussion

Although about 20% of melanomas contain RAS mutations, there are still no approved targeted therapies available for them. The challenge for targeting RAS mutant cancer lies in the complexity of targeting the RAS protein, coupled to the multiple biological properties and functional roles of RAS involved in cell growth, differentiation and survival [30]. Once activated, RAS recruits and stimulates a number of signaling pathways including MAPK and PI3K/AKT pathway. Aberrant activation of RAS leads to constitutive activation of the MAPK signal transduction pathway resulting in proliferation and promotion of tumor growth [31]. The existence of redundant feedback among RAS-MEK-ERK, PI3K/AKT pathways and intensive crosstalk among other cell survival signaling pathways, such as JNK-p38 make it extremely difficult to target RAS alone [32–34]. Various alternative strategies have been proposed, including targeting membrane localization of RAS protein by inhibiting farnesyltransferase [35], targeting upstream or downstream effectors of RAS signaling through inhibitors of EGFR, BRAF, PI3K/AKT and PLK [36,37]. Even though all these approaches hold promise, none has translated into effective clinic use. This prompts us to search alternative therapeutic strategies for RAS mutant melanoma treatment, especially to use protein kinase inhibitors in combination with therapeutics having different modes of action. One rationale is that multiple kinase inhibitors have been approved for cancer treatment, and RAS signaling pathways are intensively involved with kinase cascades [38,39].

In an attempt to search for new compounds that could partner with kinase inhibitors for inhibition of NRAS mutant melanoma [40], we combined α-Mangostin with a series of kinase inhibitors to treat the NRAS mutant melanoma cell line SK-MEL-2. Mangosteen (*Garcinia mangostana Linn*) pericarp, which contains primarily xanthones, has been used for medicinal purposes for a long time in Southeast Asia. α-Mangostin is the most abundant xanthone in mangosteen pericarp. It has been confirmed to have anti-proliferative and apoptotic effects in various types of human cancer cells at the high end of dosage [41–43]. Even though α-Mangostin has not shown an anti-cell proliferation effect in our previous human skin pigmentation studies [12], we suspected that α-Mangostin might combine with other compounds to extend its medicinal utility. We therefore performed a drug combination screening of α-Mangostin with a collection of compounds containing known-target kinase inhibitors, against SK-MEL-2, a NRAS mutant melanoma cell line.

Our primary screening results indicated that the top kinase inhibitor hits, which synergized with α-Mangostin primarily targeted three kinase clusters, i.e., Receptor Tyrosine Kinases (VEGFR, EGFR), mTOR and GSK3β. Agents targeting these enzymes included Sorafenib, Rapamycin, Torin1 and CHIR99021. Sorafenib is the first RAF kinase-targeting drug that reached clinical trials, and is an oral multi-kinase inhibitor that inhibits RAF/MEK/ERK, VEGFR, and PDGFR [44]. Sorafenib showed insufficient potency against melanoma even with BRAF mutation [45,46]. When combined with α-Mangostin, the potency of Sorefenib against SK-MEL-2 cell growth was enhanced more than 5 fold. Further clonogenic assay indicated that the combination of α-Mangostin and Sorefenib could suppress cancer cell clonogenic growth. Cell signaling analysis indicated that the cell survival pathways mediated by AKT and ERK were down regulated significantly by the combination of α-Mangostin and Sorefenib. While α-Mangostin and Sorefenib appeared to induce ER stress, it autophagy markers were diminished after exposure to the combination. Further analysis of autophagy in the context of this drug combination will be needed [20,47,48]. Since it is common for small molecule kinase inhibitors to produce eventual cancer cell resistance, it is plausible that sorafenib resistance may eventually emerge. The use of a potentiating nontoxic natural product as shown here, may improve this concern, but it remains realistic to assume that resistance may eventually arise. It will be interesting to determine whether more specific kinase inhibitors than sorafenib may produce distinct patterns of resistance.

The compounds in the family of Xanthones are known to possess a wide spectrum of pharmacologic properties, including antioxidant, anti-tumor, anti-inflammatory, and anti-bacterial activities [49]. Because of its relatively rigid chemical structures and lipophilic properties, Xanthones potentially can target a variety of cellular proteins [50]. There are several reports on the proposed cellular targets of α-Mangostin, such as CDK4 [51] and fatty acid synthase [10]. In order to search for a potential cellular target of α-Mangostin, we performed a molecular docking study using a previously reported crystal structure of RXR (PDB 3A9E). The computational data showed that α-Mangostin is compatible with the RXR’s ligand binding pocket, the common binding partner of RAR and VDR, primarily by hydrophobic interactions. α-Mangostin showed similar modes of insertion between LG100754, CF31 and its analogs into the RXR hydrophobic ligand binding cleft [15,24].

In summary, we discovered that the potential benefits of Sorafenib in combination with α-Mangostin might serve as a new therapeutic platform upon which to improve treatment of melanoma patients harboring NRAS mutations. The synthetic lethality of the α-Mangostin and Sorafenib combination might produce their effects through synergistic inhibition of MAPK/AKT signal transduction and modulation of MITF mediated cell survival pathways. Natural products have been widely studied as candidates in treatment of various cancers. There is a need to characterize promising dietary agents for chemoprevention and therapy of melanoma. Our discovery of α-Mangostin targeting RXR might provide a new mechanistic understanding of its medicinal utility. The use of these novel compounds alone or in combination therapies will hopefully provide eventual value to patients.

## Supporting Information

S1 File Supporting Information. Table A: The list of the top hit compounds that were tested. Table B: The dose dependent cell proliferation assay of α-Mangostin on human primary melanocytes. α-Mangostin shows no sign of cytotoxicity in the range of concentrations we tested, with IC50 >10 μM. Figure A: The expression of cleaved-PARP and PARP. The expression of cleaved-PARP and PARP were analyzed by western blot in SK-MEL-2 cell lines exposed to Sorafenib (2 μM) in either the presence or absence of α-Mangostin (2 μM) for 8 hours. The expression of cleaved-PARP was increased with the combination of Sorafenib and a-Mangostin.
(PPTX)
