# Supplementary material for: Inhibition of Cell Proliferation in an NRAS Mutant Melanoma Cell Line by Combining Sorafenib and α-Mangostin
Source: PLoS One. 2016 May 6;11(5):e0155217. doi: 10.1371/journal.pone.0155217 (PMC4859503; doi:10.1371/journal.pone.0155217)
Supplement: S1 File — Table A: The list of the top hit compounds that were tested. Table B: The dose dependent cell proliferation assay of α-Mangostin on human primary melanocytes. α-Mangostin shows no sign of cytotoxicity in the range of concentrations we tested, with IC50 >10 μM. Figure A: The expression of cleaved-PARP and PARP. The expression of cleaved-PARP and PARP were analyzed by western blot in SK-MEL-2 cell lines exposed to Sorafenib (2 μM) in either the presence or absence of α-Mangostin (2 μM) for 8 hours. The expression of cleaved-PARP was increased with the combination of Sorafenib and a-Mangostin. (PPTX) [file pone.0155217.s001.pptx]

## Slide 1
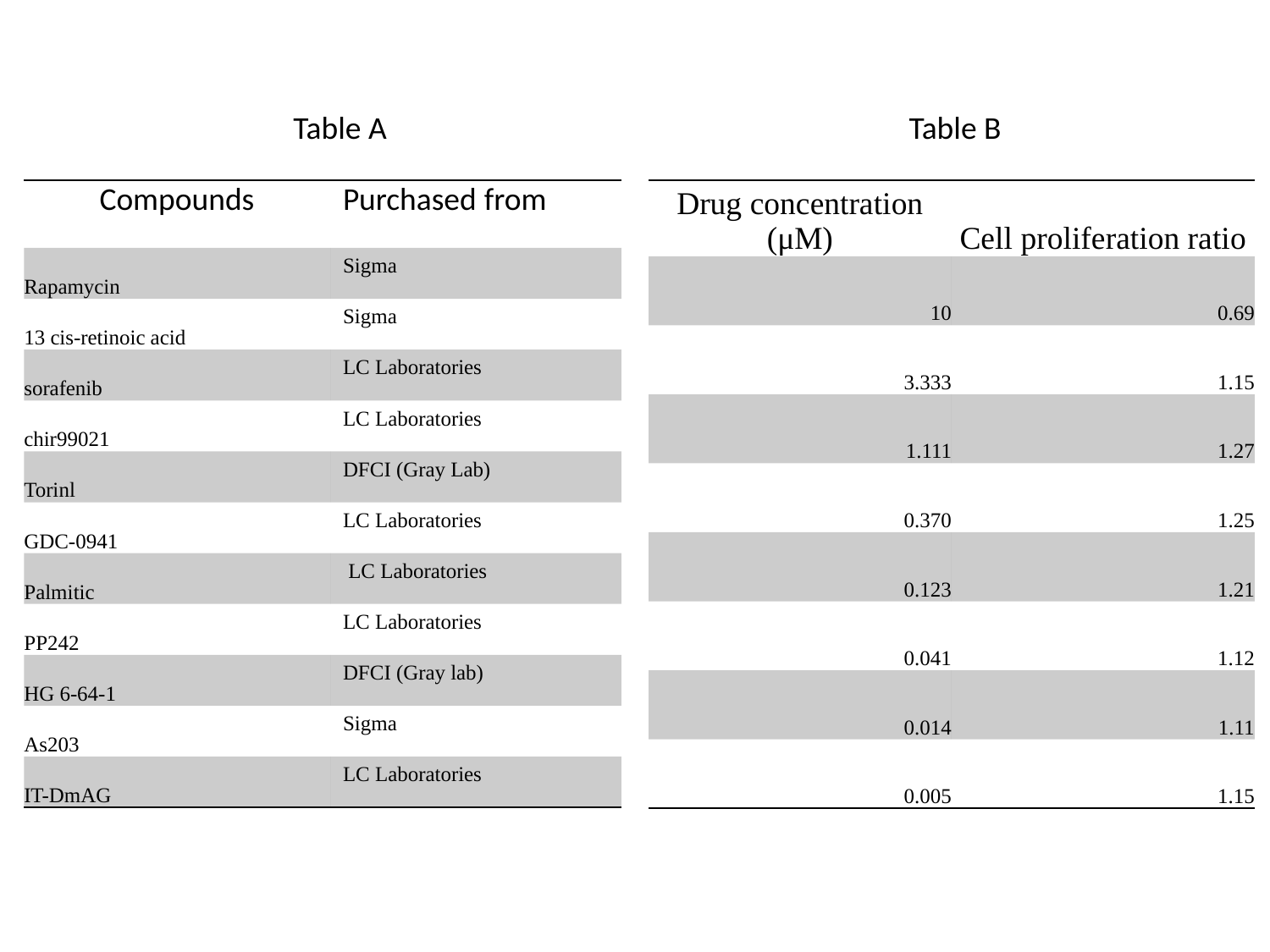

Table A
Table B
| Compounds | Purchased from |
| --- | --- |
| Rapamycin | Sigma |
| 13 cis-retinoic acid | Sigma |
| sorafenib | LC Laboratories |
| chir99021 | LC Laboratories |
| Torinl | DFCI (Gray Lab) |
| GDC-0941 | LC Laboratories |
| Palmitic | LC Laboratories |
| PP242 | LC Laboratories |
| HG 6-64-1 | DFCI (Gray lab) |
| As203 | Sigma |
| IT-DmAG | LC Laboratories |
| Drug concentration (μM) | Cell proliferation ratio |
| --- | --- |
| 10 | 0.69 |
| 3.333 | 1.15 |
| 1.111 | 1.27 |
| 0.370 | 1.25 |
| 0.123 | 1.21 |
| 0.041 | 1.12 |
| 0.014 | 1.11 |
| 0.005 | 1.15 |

## Slide 2
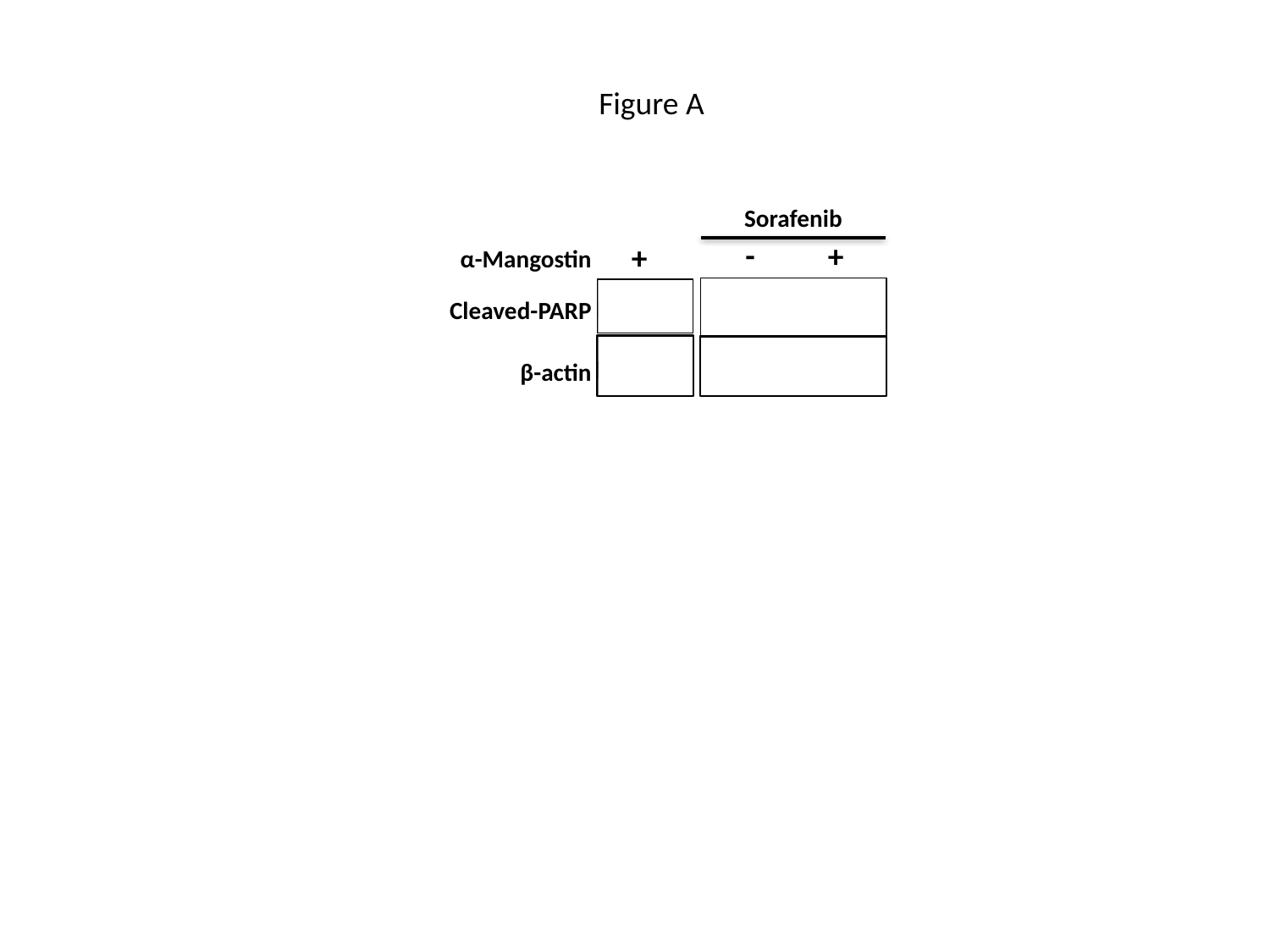

Figure A
Sorafenib
-
+
+
Cleaved-PARP
β-actin
α-Mangostin
